# Effects of Solar Radiation on Leaf Development and Yield of Tuberous Roots in Multilayered Sweet Potato Cultivation

**DOI:** 10.3390/plants12020287

**Published:** 2023-01-07

**Authors:** Takahiro Suzuki, Masaru Sakamoto, Hiroshi Kubo, Yui Miyabe, Daisuke Hiroshima

**Affiliations:** 1 Faculty of Biology-Oriented Science and Technology, Kindai University, Wakayama 649-6493, Japan; 2 Japan Sewage Works Agency, Higashiku, Nagoya 461-0025, Japan; 3 Water Agency Inc., Hamamatsu 435-0016, Japan

**Keywords:** sweet potato, solar radiation, photosynthetic yield, multilayered cultivation, polyphenol

## Abstract

The purpose of this study was to develop a novel method to dramatically improve the production efficiency of sweet potatoes (*Ipomoea batatas* (L.) Lam.) by elucidating the effect of solar radiation stress on the growth of sweet potato in a multilayer cultivation system. Twenty-five pots planted with sweet potato vine seedlings were arranged in three layers and cultivated for 160 days while supplying liquid fertilizer to the root zone. While solar radiation in the middle and lower layers decreased to 69% and 45% of that in the upper layer, respectively, the yield of tuberous roots was 0.89 kg/pot in the upper layer, 0.79 kg/pot in the middle layer, and 0.66 kg/pot in the lower layer. As a result, the productivity of tuberous roots reached 10.5 kg/m^2^, 4.4 times that of conventional farming. On the other hand, the amounts of leaves and stems increased in the lower layer than in the upper layer, and the biomass energy yield (photosynthetic efficiency) was 2.8% in the upper layer, 3.7% in the middle layer, and 5.1% in the lower layer. Leaves in the lower layer with less solar radiation had a lower polyphenol content and increased the amounts of low-brightness leaves. In contrast, the upper leaves were found to contain more polyphenols and have brighter, smaller leaves. These results suggest that the yield can be further increased by optimizing solar radiation stress by using the multilayer cultivation method.

## 1. Introduction

Sweet potato (*Ipomoea batatas*) is an important root vegetable cultivated in temperate and tropical zones, especially in Asia and Africa [1]. Storage roots of sweet potato contain relatively high amounts of carbohydrates that support the demand for food in developing countries [2,3]. In recent years, sweet potato has been also evaluated as a candidate for biofuel production [4,5].

Sweet potatoes store a lot of starch in their tuberous roots, and under the climatic conditions in Japan, calorie production per area reaches 3170 kcal/m^2^ with a normal yield of 2.4 kg/m^2^, compared to 1900 kcal/m^2^ for rice (yield 0.54 kg/m^2^), 1500 kcal/m^2^ for wheat (0.44 kg/m^2^), and 1400 kcal/m^2^ for sweet corn (1.5 kg/m^2^) [6]. In addition, the leaves and stems of sweet potatoes are also consumed as edible vegetables [7]. Such edible biomass can be converted to methane in high yield by anaerobic digestion [8,9]. Therefore, mass production of sweet potatoes as a resource crop to supply biomethane for use in power generation and heat is gaining attention as an alternative to fossil fuels.

Although woody biomass is generally considered a carbon-neutral resource, annual growth in Japan’s 250,500 km^2^ of forest is about 68 million m^3^ [10], and if we assume that 50% of solids have 22 MJ/kg of heat, the yield of photosynthesis is very low, at about 0.06% of annual solar radiation (assuming an average of 5000 MJ/m^2^ y).

The current total energy quantity of 5.3 billion m^3^ of forest biomass in Japan [10] is estimated to be 58 trillion MJ, equivalent to 3 years of fossil fuel consumption in Japan [11]. In other words, if all fossil fuels are replaced with domestic woody fuels, they will run out in 3 years, and even if planted there, it will take more than 50 years to regenerate.

Moreover, the thermal power generation efficiency of woody fuel is low, ranging from 25 to 30% [12], which is lower than that of coal fuel, which has a power generation efficiency of 43% [11]. As a result, by converting the fuel from coal to woody biomass, CO_2_ emissions per unit of electricity generated increased to 140%–170%. In other words, woody biomass in Japan is a fuel that can neither achieve carbon neutral nor reduce greenhouse gases.

On the other hand, all of the CO_2_ emitted in the process of producing biogas from sweet potatoes and consuming biomethane can be absorbed every year in the process of regenerating sweet potato fuel by the high photosynthetic yields. This allows for sweet potato biomethane to remain carbon neutral, so no amount of combustion will increase the amount of CO_2_ stored in the atmosphere. Therefore, replacing natural gas, coal, and oil with this biomethane is expected to significantly reduce CO_2_ emissions from fossil fuels.

In Germany, biogas power generation using dent-corn and other resource crops is widespread, and the share of biogas power generation in the total electricity has increased to 6% [13]. On the other hand, 66% of Japan’s 377,727 km^2^ land area is mountainous, with only 43,490 km^2^ of agricultural land, or 26% of that of Germany. In addition, half of the agricultural land is devoted to rice cultivation, which is a staple food, making it difficult to mass produce resource crops.

Therefore, in order to mass produce resource crops that can replace fossil fuels, it is necessary to develop new cultivation methods that dramatically increase the biomass production per unit area. From this perspective, we studied the cultivation methods that enhance the photosynthetic efficiency of sweet potatoes [14].

Thus far, the yield of tuberous roots of sweet potatoes grown under rhizosphere irrigation has been found to reach 1.5 kg when grown in 21 cm top diameter vinyl pots filled with 4.5 L of horticultural soil [15]. The results suggest an increase in productivity far beyond the average yield of 2.4 kg/m^2^ [6,16] for open-field cultivation and indicate that soil volume and land area are not essential for sweet potato production efficiency.

In addition, the photosynthetic efficiency of sweet potatoes tended to decrease under strong sunlight conditions [17,18,19,20], suggesting that solar energy cannot be fully utilized by conventional farming methods.

Based on these growth characteristics, the authors devised a multilayer cultivation method to reduce the intensity of solar radiation by increasing the spread area of leaves and decreasing the angle of the light reception of leaves, conducted cultivation tests under solar radiation, and found that the photosynthetic efficiency per field greatly improved.

Therefore, the purpose of this study was to clarify the effect of solar radiation stress on the growth of sweet potato and to develop a new method for dramatically improving production efficiency.

## 2. Results

### 2.1. Insolation Rate in Three-Layers Cultivation System

**Figure 1:**
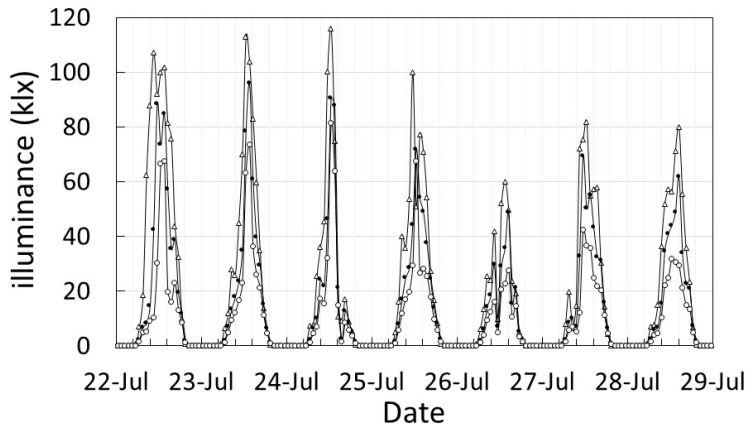
Measurement record of illuminance for one week from 22 July in the three-layer cultivation system. Symbols, △: Top, ●: M_in_, 〇: B_in_.

Figure 1 shows the results of the Top (top layer), M_in_ (inside of middle layer) and B_in_ (inside of bottom layer) illuminance measurements in the three-layer cultivation system for a week from 22 July, which is known as the hottest week with the strongest intensity of ultraviolet rays (UV) in a typical year of the meteorological statistics.

In the same way, the relative values of each layer were obtained by integrating the illuminances measured from July to August during the peak growing season. Using the integrated value of Top as 100%, the solar radiation (SR) for each layer was 85% for M_out_ (outside of middle layer), 69% for M_in_, 73% for B_out_ (outside of bottom layer), and 45% for B_in_. The illuminance on the east side in the morning and on the west side in the afternoon was the same as the Top, so the cumulative illuminance was higher on the outside than on the inside. These relative values were used to calculate the amount of SR in each layer. As a result, the estimated SR in the upper layer was 2480 MJ/m^2^, 2108 MJ/m^2^ in M_out_, 1711 MJ/m^2^ in M_in_, 1810 MJ/m^2^ in B_out_, and 1116 MJ/m^2^ in B_in_.

### 2.2. Productivities of Sweet Potatoes in Three-Layer Cultivation Systems

**Figure 2:**
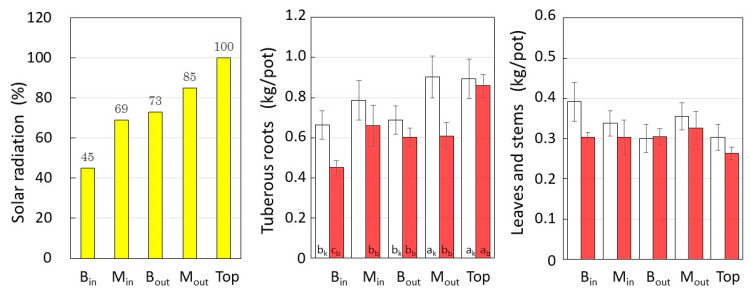
The amount of solar radiation in each layer, and the fresh weight of tuberous roots, leaves, and stems per pot harvested in each layer. Symbols: □: *Suikenkintoki*, ■: *Beniharuka*. Data are means and error bars represent standard error of the mean. Different letters indicate statistically significant differences between cultivation layers (*p* < 0.05).

Figure 2 shows the estimated SR, the average fresh weight of tuberous roots (TR), and leaves and stems (LS) per pot harvested in each layer. The yield of *Beniharuka* (BH) was 453 g/pot for M_in_ at 45% SR and 859 g/pot for the Top layer. On the other hand, for *Suikenkintoki* (SK), a peak of 903 g/pot was obtained at 83% SR of M_out_, while it was almost equivalent to 893 g/pot by 100% SR of Top. The yield of SK was reduced to 662 g/pot in M_in_ at the lowest SR.

In contrast, both cultivars had smaller amounts of leaves and stems in the top layer at 264 g/pot for BH and 303 g/pot for SK, and the amount increased significantly to 391 g/pot for SK in the lower layer with lower SR.

**Figure 3:**
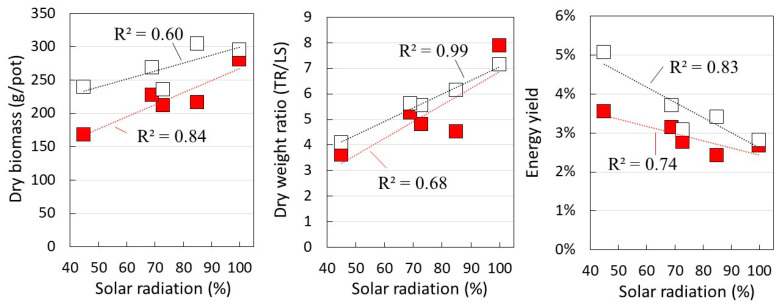
Dry biomass weight per pot, the dry weight ratio of tuberous roots (TR) to leaves and stems (LS), and energy yield, in each solar radiation rate. Symbols: □: *Suikenkintoki*, ■: *Beniharuka*. The data are the mean values, the dotted lines are the approximate lines determined by the least-squares method, and R^2^ is the coefficient of determination of the approximate line, indicating the degree of correlation between each element.

Figure 3 shows the dry biomass weight per pot, the dry weight ratio of tuberous roots (TR) to leaves and stems (LS), and energy yield at each level of solar radiation. In both cultivars, the amount of dry biomass increased as the amount of solar radiation increased. The dry weight ratio of TR/LS also increased with a higher solar radiation. These results indicate that the higher the amount of solar radiation, the more likely the amount of tuberous roots is to increase.

On the other hand, the energy yield based on the amount of SR in each layer increased in the lower layer with less solar radiation. The yield of SK rose to 5.1% at M_in_ with 45% solar radiation, while the Top layer was 2.8%. Similarly for BH, M_in_ yield increased to 3.5%, while the M_out_ and Top yields were lower at 2.7% and 2.4%, respectively.

These results clearly show that the three-dimensional distributed use of sunlight increased the biomass yield of sweet potato per field area. On the other hand, even in three-layer cultivation, the top layer showed that some of the solar radiation was wasted.

### 2.3. Photosynthetic Efficiency in Three-Layers Cultivation

**Figure 4:**
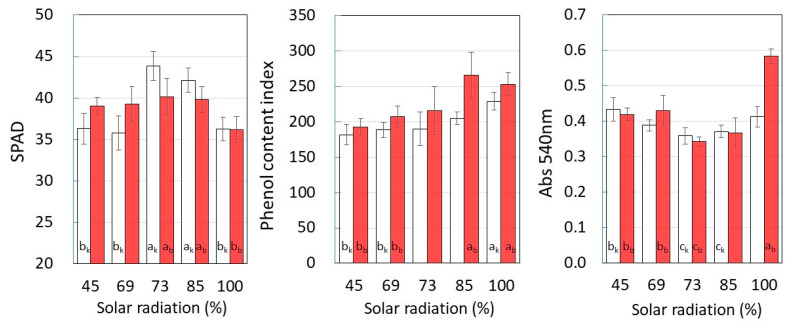
Chlorophyll content (SPAD), total phenol content index, and 540 nm absorbance of the leaf extract in each solar radiation rate. Symbols: □: *Suikenkintoki*, ■: *Beniharuka*. Data are the means and error bars represent the standard error of the mean. Different letters indicate statistically significant differences between cultivation layers (*p* < 0.05).

Figure 4 shows the chlorophyll content (SPAD), total phenolic content index, and absorbance at 540 nm of leaf extract at each level of solar radiation. In both cultivars, it was revealed that the chlorophyll content reached a maximum in B_out_ and the top layer decreased. In contrast, the phenol content increased with higher solar radiation.

Polyphenols are known to absorb and attenuate ultraviolet rays (UV) to prevent the degradation of chlorophyll by UV [21]. For this reason, plants need to produce more polyphenols at higher levels of solar radiation, and UV is known to enhance polyphenol production [22,23]. The results in the figure also show that the polyphenol content increased with UV, depending on the amount of solar radiation in each layer.

On the other hand, it was supposed that the Top layer was insufficiently protected from UV rays by polyphenols, which increased the degradation of chlorophyll. These results suggest that a high solar radiation rate decreases photosynthetic efficiency because chlorophyll is degraded by UV.

The figure also shows the absorbance at 540 nm. The absorption at 540 nm is thought to depend on the amounts of carotenoids [24] and flavonoids including anthocyanins [25,26]. In plants, yellowish carotenoids are found in chloroplast membranes [27] and function to absorb and reflect excess light and UV radiation, and to inhibit the production of reactive oxygen species. Both are thought to increase production to reduce the degradation of chlorophyll by UV, but the details of their effects on sweet potato leaves are unclear. The figure shows a significant increase in absorption at 540 nm in the Top layer with high polyphenol content and low SPAD value, suggesting a higher content of polyphenols and carotenoids compared to chlorophyll.

Degradation of chlorophyll may have made the carotenoid pigments easier to extract, but in any case, degradation of chlorophyll by the strong UV in the upper layer may have reduced the photosynthetic efficiency.

**Figure 5:**
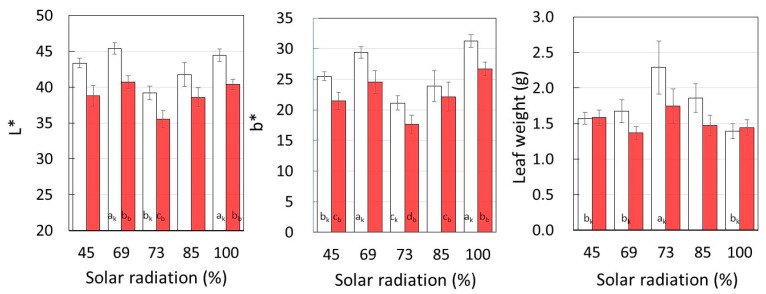
Leaf surface lightness (L*) and yellowness (b*) in each solar radiation, and individual leaf weight. □: *Suikenkintoki*, ■: *Beniharuka*. Data are the means and error bars represent standard error of the mean. Different letters indicate statistically significant differences between cultivation layers (*p* < 0.05).

Figure 5 shows the average values of the leaf surface brightness (L*), yellowness (b*), and individual leaf weights for each insolation. In the Top layer, L* and b* were higher and the leaf size was smaller, but when the light intensity decreased to 73% in B_out_, L* and b* decreased and the leaf growth increased. These results indicate that strong sunlight conditions increase leaf L* and b* and decrease leaf size.

On the other hand, the leaf L* and b* and leaf size of the inner M_in_ (69%) were similar to those of Top, while that of B_in_ (45%) decreased the leaf brightness. These results suggest that there were other factors that limited the growth of sweet potatoes on the inner side of the multi-layered shelves besides the rate of sunlight.

Comparing the results in Figure 4 and Figure 5, it can be seen that SPAD and L* and b* including the inside of the multilayer shelves showed the opposite trend, with leaves with high chlorophyll content having a lower amount of reflected light due to increased light absorption. In contrast, leaf size and SPAD showed the same trend, indicating that leaves with high chlorophyll content increased the size to absorb light efficiently under low sunlight conditions.

**Figure 6:**
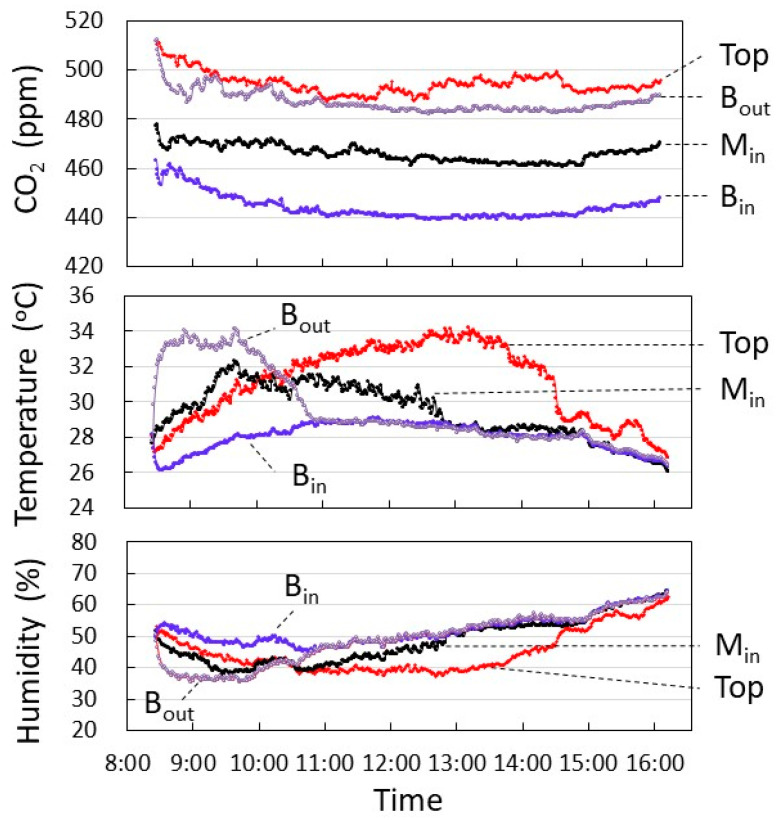
CO_2_ concentration, temperature, and humidity of each layer measured in September. Data on clear days from 24–29 September were averaged to show time changes.

Figure 6 shows the CO_2_ concentration, temperature, and humidity of each layer measured in September. Data on clear days from 24–29 September were averaged to show the time changes. CO_2_ concentration at B_out_ was maintained within 15 ppm of Top, while the inside M_in_ was 30 to 40 ppm lower than Top, and B_in_ was a further 20 ppm lower.

It also showed lower temperatures than Top after 10:30 a.m. in M_in_ and B_in_. These results suggest that the three-dimensional dense planting condition of sweet potato reduces the CO_2_ concentration in the middle and lower layers of the growth shelf, and that low temperature also affects the suppression of leaf growth and chlorophyll accumulation, resulting in low SPAD and high L* and b*.

**Figure 7:**
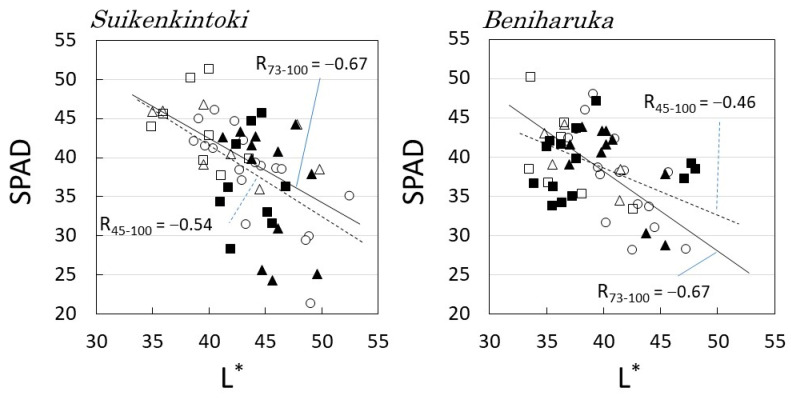
Relationships between leaf lightness (L*) and chlorophyll content (SPAD) in each solar radiation rate. Symbols: 〇: Top (SR = 100%), △: M_out_ (85%), □: B_out_ (73%), ▲: M_in_ (69%), and ■: B_in_ (45%). The approximate lines were determined by the least-squares method, and R_45–100_ denotes the correlation coefficient for all data, and R_73–100_ denotes the correlation coefficient for data excluding M_in_ and B_in_.

The relationship between the chlorophyll content (SPAD) of each layer and the leaf lightness (L*) is shown in Figure 7. In both cultivars, leaves with lower L* tended to have higher SPAD. This indicates that leaves with higher SPADs have more chlorophyll, which absorbs light, and lower brightness due to less reflected light.

The figure also shows the correlation coefficient between SPAD and L* and the approximate line obtained by the least-squares method. R_45-100_ denotes the correlation coefficient for all data, and R_73-100_ denotes the correlation coefficient for data excluding M_in_ and B_in_. Both varieties showed a high negative correlation with R_73-100_ of −0.67, indicating that under conditions where CO_2_ concentration and temperature are well maintained, the upper part with higher solar radiation has higher L* and lower SPAD.

From these results, it can be concluded that leaves in the low layer with low solar radiation increased the absorption rate of sunlight by decreasing L* and increasing the chlorophyll content, thus increasing the yield of photosynthesis.

**Figure 8:**
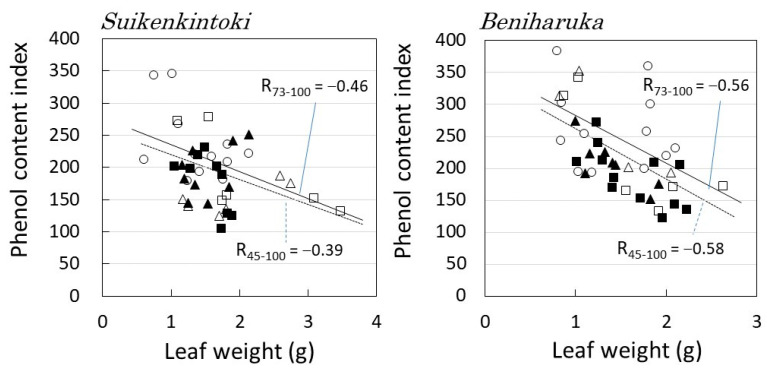
Leaf weight and polyphenol content. Symbols are the same as in Figure 7. The approximate lines were determined by the least-squares method, R_45–100_ denotes the correlation coefficient for all data, and R_73–100_ denotes the correlation coefficient for data excluding M_in_ and B_in_.

Figure 8 shows the relationship between the weight of individual leaves and their phenolic content. In both cultivars, the larger the leaf size, the lower the phenol content. It can also be seen that the upper layer with high solar radiation has many small leaves with high phenol content, while the lower layer has many large leaves with low phenol content.

These results suggest that the upper layer with high insolation carries out photosynthesis while increasing light reflectivity through polyphenols and carotenoid dyes and reducing light stress by reducing leaf size so as to not absorb excess light.

In contrast, it was considered that leaves with low solar radiation increased the amount of light absorbed by expanding the leaf area and decreasing the reflectance, thereby making efficient use of less light.

**Figure 9:**
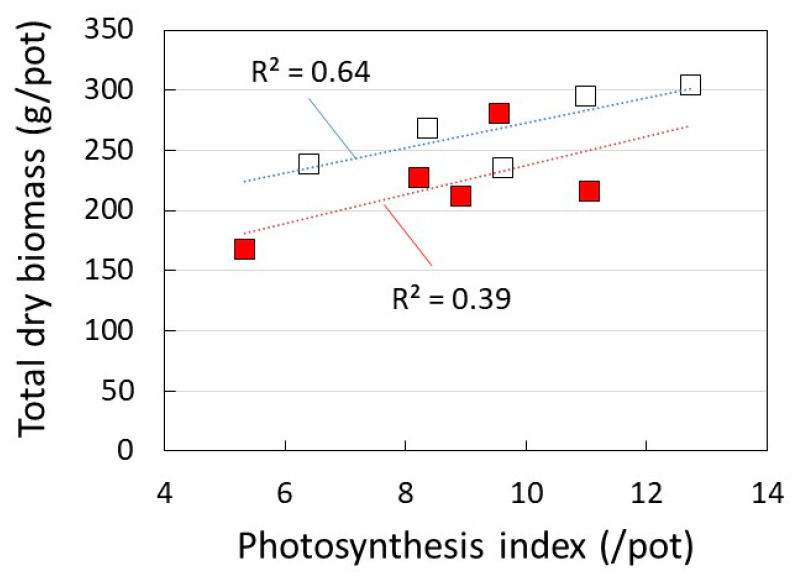
Relationship between the photosynthesis index [SPAD × leaves and stems (kg/pot) × solar radiation (%)] and total dry biomass production (tuberous roots, leaves and stems) in the three-layer cultivation of sweet potato. Symbols: □: *Suikenkintoki*, ■: *Beniharuka*.

Figure 9 shows that the total dry biomass production of each layer increased against the photosynthetic reaction rate index multiplied by three factors: chlorophyll content, leaf and stem volume (kg/pot), and solar radiation (%). In the three-layer cultivation method, it was concluded that the photosynthetic yield increased compared to the upper layer because the amounts of leaves and chlorophyll increased as the solar radiation including strong ultraviolet rays decreased at the lower layer.

Based on the above results, the effects of the three-layer cultivation method in increasing the production of sweet potato biomass were summarized in Table 1. The upper row of the table shows the fresh weight of tuberous roots and stems and leaves, and the lower row shows the dry weight.

In a mass production system combining a number of three-layer cultivation systems, the productivity was estimated to be 10.5 kg/m^2^ for SK and 8.6 kg/m^2^ for BH from the results of this study. As a result, SK is expected to be 4.4 times more productive and BH 3.7 times more productive than conventional farming at 2.4 kg/m^2^.

On the other hand, the energy yield of the dried biomass of stems, leaves, and tuberous roots was calculated to be 2.5% for SK and 2.0% for BH relative to the insolation energy. Conventional sweet potato production yields 2–3 kg/m^2^ of stems and leaves for every 2.4 kg/m^2^ of tuberous roots, with an estimated total dry weight of 1.0 kg/m^2^ (17 MJ/m^2^) and a photosynthetic yield of 0.7% (=17 MJ/2480 MJ). Therefore, it was shown that the three-layer cultivation method could increase the photosynthetic efficiency of sweet potatoes to three times that of the conventional method.

However, compared with the highest yield at 5.1% of effective solar radiation in B_in_, as shown in Figure 3, the actual photosynthetic yield was only about half, ranging from 2.0% to 2.5%. Further productivity gains are therefore expected by improving methods to increase the efficiency of the distributed use of solar radiation.

## 3. Discussion

Due to the low light saturation of sweet potatoes, the search for varieties that enhance photosynthetic efficiency and research to improve the yield are actively underway. In reality, however, the average yield of sweet potatoes on agricultural land has not increased in half a century.

On the other hand, the calorie productivity of sweet potatoes was nearly twice as high as that of wheat, corn, and rice, which have higher light saturation points than sweet potatoes, and the same is true overseas. Therefore, while sweet potatoes may be wasting some solar energy in the summer, when the sun’s intensity increases, they are very capable of producing food and fuel. Therefore, we investigated the effect of solar radiation intensity on the photosynthetic reaction of sweet potato, and hypothesized that if the solar radiation intensity is optimized, the photosynthetic yield could be further enhanced, and we conducted this study to test this hypothesis.

As a result, even when the insolation rate was 45%, the dry biomass production of both stems and potatoes reached 81% for *Suikenkintoki* and 60% for *Beniharuka* compared to the top layer. The effects of solar radiation stress on sweet potato, which is used as a staple food source worldwide, became partially apparent, resulting in a tuberous root production of 10.5 kg/m^2^ and 13,900 kcal/m^2^ in the three-layer cultivation. This was 4.4 and 7.3 times higher than the production of sweet potato and rice under the conventional farming described in the introduction, respectively.

As the world’s population continues to grow beyond 8 billion, there is strong concern that deforestation for agricultural land expansion will continue to increase, further accelerating global warming. However, the results of this study are the first in the world to demonstrate that food production can be significantly increased without increasing the area of agricultural land.

The photosynthetic yield of sweet potato biomass reached 2.0% to 2.5%. This is 33–41 times more efficient than the current 0.06% photosynthetic yield of Japanese forests. In other words, it became clear that the same area could absorb 33 to 41 times more CO_2_ if the forest was converted to a multilayer cultivation field for sweet potatoes.

On the other hand, when the amount of solar radiation was low, the amounts of leaves and stems increased relative to the tuberous roots. To increase biomass productivity, it is desirable to grow tuberous roots efficiently with fewer leaves, so the growing conditions of sweet potatoes need to be further investigated.

Lin et al. investigated the color and photosynthetic efficiency of sweet potato leaves and reported that green leaves accumulated more biomass, while yellow leaves had less biomass production and increased heat release [28]. These results are consistent with the above results, indicating that when the solar radiation intensity is high, photosynthetic efficiency is reduced by producing more yellow pigments such as flavonoids and polyphenols than chlorophyll.

The effect of solar radiation stress such as UV irradiation on tuberous root enlargement in sweet potato is largely unknown and appears to vary among varieties [29,30]. However, the results presented here suggest that solar stress may have promoted the enlargement of tuberous roots, while leaf light use decreased with increasing solar radiation intensity.

Sweet potatoes barely bloom and produce no seeds in the warm, humid climate of spring and summer in Japan. For this reason, the accumulation of tuberous roots is considered to be one of the important means of the survival of sweet potatoes during the cold and dry season from autumn to winter. It is also known that in ordinary farming methods, high amounts of nitrogen fertilizer overgrow stems and leaves, reducing crop yields without enlarging tuberous roots.

The common cultivation know-how of sweet potato farmers suggests that the upper layer promotes tuberous root enlargement to avoid the risk of survival due to strong UV stress, while the middle and lower layers prioritize the growth of stems and leaves with the UV stress of sunlight relieved. However, it is difficult to elucidate the effect of solar UV stress on the enlargement of tuberous roots by the present experimental results alone. Further research on solar radiation and UV stress is expected to significantly improve the production efficiency of biofuel resources as well as food crops.

## 4. Materials and Methods

### 4.1. Three-Layer Cultivation System

Sweet potato (*Ipomoea batatas*) cultivars *Suikenkintoki* (SK) and *Beniharuka* (BH) were used. Regarding the sweet potato seedlings, commercial vine seedlings were purchased in the same way as general farmers, planted in each pot in early June, grown for 160 days and harvested in early November.

**Figure 10:**
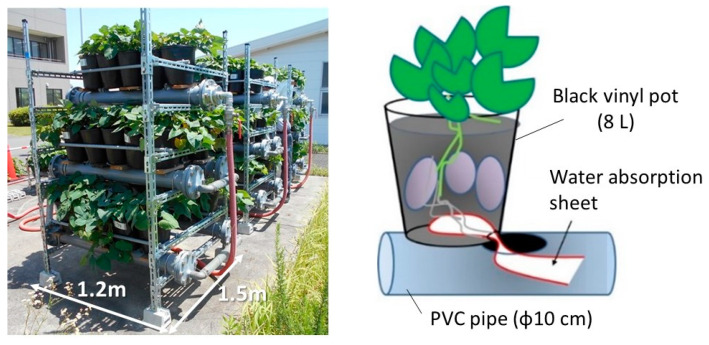
The three-layer cultivation system with rhizosphere irrigation.

Figure 10 shows the three-layer cultivation system used in this study. Five plastic pots (capacity; 8 L) were placed on an irrigation vinyl chloride (PVC) pipe (inner diameter; 10 cm) and each pot was filled with 7 L of gardening soil to plant the sweet potato seedlings. This set of five pots was placed in a row in the upper layer and two rows in the middle and lower layers for three-layer cultivation. The occupied area of the three-layer–25 pots cultivation system was 1.8 m^2^.

Using the method introduced in a previous paper [14,15], a water-absorbing sheet was inserted into the PVC pipe from the bottom of the pot and guided so that the thin roots of the sweet potatoes extended into the pipe containing water and liquid fertilizer.

The liquid fertilizer for the gardening of flowers and vegetables was 500 times diluted and supplied once a day through the PVC pipe. The approximate concentrations of the diluted fertilizer were: N: 120 mg/L, H_3_PO_4_: 200 mg/L, K: 100 mg/L, Mg: 1 mg/L, Mn: 0.02 mg/L, B: 0.01 mg/L.

### 4.2. Solar Radiation Rates in a Three-Layer Cultivation System

**Figure 11:**
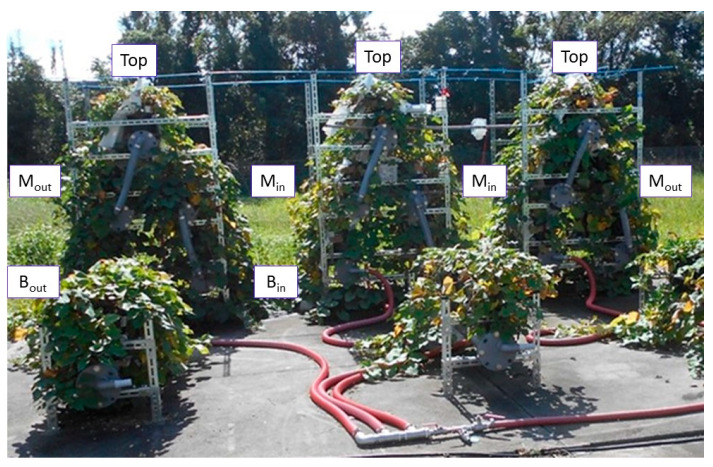
Position notations of the three-layer cultivation system: Top, top layer, M_out_, middle layer outside, M_in_, middle layer inside, B_out_, bottom layer outside, B_in_, bottom layer inside. The monolayer cultivation system in the foreground is for comparison.

Figure 11 shows the sweet potato cultivation in the three-layer cultivation system and the position of each layer. The top layer is Top, the outside of the middle layer is M_out_, the inside of the middle layer is M_in_, the outside of the lower layer is B_out_, and the inside of the lower layer is B_in_. A monolayer culture system was also used for comparison.

The illuminance of each layer was measured using an illuminance UV recorder (TR Type-74 Ui, T&D, Tokyo, Japan) during July–August, when sweet potato grows actively, and the cumulative values were compared to the Top layer. The solar radiation ratio (SR) of each layer was given as a relative value compared to the Top layer. The CO_2_ concentration, temperature, and humidity of each layer were measured using a CO_2_ datalogger (TR Type-76 Ui, T&D, Tokyo, Japan).

The cumulative value of solar radiation at the top during the entire 160 d cultivation period was set at 2480 MJ/m^2^ by referring to measurements taken at the nearest meteorological observatory (Shizuoka City). Each SR was then multiplied by 2480 MJ/m^2^ to calculate the cumulative solar radiation inside and outside the middle and lower layers.

### 4.3. Photosynthetic Efficiency in Three-Layer Cultivation

Harvested tuberous roots, leaves, and stems were dried at 90 °C for one day and the dry matter content measured. The unit heat of dry matter was set at 17 MJ/kg referring to previous reports [31]. To analyze the effect of SR on leaf photosynthetic efficiency, the rate of energy conversion to biomass was calculated by dividing the thermal energy stored in the tuberous roots and stems by the cumulative amount of solar radiation in each layer. The heat yield of each layer was converted to productivity at 1 m^2^ by the equation of “harvested quantity kg/pot × 25 pots/1.8 m^2^ × 17 MJ/kg”, and the energy yield for the estimated solar radiation (SR × 2480 MJ/m^2^) of each layer was obtained.

To analyze the photosynthetic efficiency during the active growing season, three leaves of each size (S = 4–10 cm, M = 10–13 cm, L = 13 cm or more) were collected from each pot 90 days after planting, and each leaf was weighed. The lightness (L*), redness (a*), and yellowness (b*) of each leaf were measured using a color reader, CR-10 (Konica Minolta Co. Ltd., Minato, Tokyo) [28]. In addition, the relative chlorophyll content by SPAD-502 (Konica Minolta, Inc.) was determined [32].

To investigate the effect of SR on the polyphenol content of leaves, polyphenols were extracted from leaf segments cut to 1 cm^2^ with 50% aqueous methanol and measured for absorbance at 765 nm using the Folin–Ciocalteu method previously reported [33,34]. The effects of SR were analyzed using the “765 nm absorbance × dilution ratio” as the phenol content index per leaf area.

### 4.4. Statical Analysis

Statistical analysis was performed using the statistical analysis tool in Microsoft Excel 2019. The mean value and standard error of the opposing elements were obtained using each solar radiation rate (position of the shelf) as a conditional element was indicated (Figure 2, Figure 4 and Figure 5). Analysis of variance (ANOVA) was also applied to the resulting data. The significance of the differences between the condition elements was determined by a t-test between the two elements assuming unequal variance, and groups of *p* < 0.05 significant differences were indicated by different letters in the figures.

Figure 3 and Figure 9 show the correlations between the conditional elements and the mean values of opposing elements using the approximate lines obtained by the least-squares method and the coefficient of determination (R^2^). Figure 7 and Figure 8 showed an approximate line and a correlation coefficient (R) obtained by the least-squares method for the correlation between the conditional element and the opposing element.

## 5. Summary

This study showed that reducing solar radiation stress by using a three-layered cultivation method of sweet potato increased the yield 4.4 times higher than conventional cultivation methods. In addition, to clarify the effects of solar radiation stress, we investigated the pigments, polyphenols, and shapes involved in the protection of leaves from UV stress. As the result, it was found that when sufficient CO_2_ and fertilizers were available, the leaf size tended to decrease and the polyphenol content, lightness, and yellowness increased under intense solar radiation.

From these results, it can be concluded that sweet potatoes, which have low light saturation points and photosynthetic yields under conventional farming methods, block some ultraviolet and visible light with polyphenols and pigments to mitigate strong photooxidation, and reduce leaf size so as to not absorb extra light.

Furthermore, from these analyses, it was concluded that the increase in photosynthetic yield by the multilayer cultivation method was due to the increase in light absorption due to the three-dimensional increase in leaf area and the relaxation of UV stress.

## Figures and Tables

**Table 1:** Productivity of sweet potatoes in three-layer cultivation and energy yield to the solar radiation.

		Tuberous Roots		Leaves and Stems		Total Biomass	
Fresh biomass		SK	BH	SK	BH	SK	BH
Top	g-fm/pot	893	859	303	264	1196	1122
M_in_	g-fm/pot	786	659	338	303	1124	962
B_in_	g-fm/pot	663	453	391	303	1054	756
Total yield *	kg-fm/25 pots	19.0	15.4	8.8	7.4	27.8	22.8
Productivity	kg-fm/m^2^	10.5	8.6	4.9	4.1	15.4	12.7
Dry biomass		SK	BH	SK	BH	SK	BH
Top	g-dm/pot	259	249	36	32	295	281
M_in_	g-dm/pot	228	191	41	36	269	228
B_in_	g-dm/pot	192	131	47	36	239	168
Total yield *	kg-dm/25 pots	5.5	4.5	1.1	0.9	6.6	5.4
Energy yield	MJ-dm/MJ-solar	2.1%	1.7%	0.4%	0.3%	2.5%	2.0%

Total yield * = mean yield of Top × 5 pots + mean yield of M_in_ × 10 pots + mean yield of B_in_ × 10 pots.

## Data Availability

The data presented in this study are available on request from the corresponding author.

## References

[1] Petsakos A., Prager S.D., Gonzalez C.E., Gama A.C., Sulser T.B., Gbegbelegbe S., Kikulwe E.M., Hareau G. (2019). Understanding the consequences of changes in the production frontiers for roots, tubers and bananas. Glob. Food Secur..

[2] Woolfe J. (1992). Sweet Potato: An Untapped Food Resource.

[3] Pimentel D., Doughty R., Carothers C., Lamberson S., Bora N., Lee K. (2002). Energy inputs in crop production in developing and developed countries. Food Security and Environmental Quality in the Developing World.

[4] Koçar G., Civas N. (2013). An overview of biofuels from energy crops: Current status and future prospects. Renew. Sustain. Energy Rev..

[5] Lareo C., Ferrari M.D. (2019). Sweet potato as a bioenergy crop for fuel ethanol production. Perspectives and Challenges. Bioethanol Production from Food Crops.

[6] Crop Survey (Rice, Barley, Beans, Sweet Potatoes, Forage Crops and Industrial Crops), Annual Statistics on Agriculture, Forestry and Fisheries FY2021 (in Japanese), Ministry of Agriculture, Forestry and Fisheries, Tokyo, Japan, 2022. https://www.maff.go.jp/j/tokei/kouhyou/sakumotu/sakkyou_kome/index.html#y17.

[7] Eguchi T., Moriyama S., Miyama I., Yoshida S., Chikushi J. (2007). A hydroponic method suitable for tops production of a sweetpotato cultivar ‘Suioh’. J. Sci. High Technol. Agric..

[8] Montoro S.B., Lucas Jr J., Santos D.F.L., Costa M.S.S.M. (2019). Anaerobic co-digestion of sweet potato and dairy cattle manure: A technical and economic evaluation for energy and biofertilizer production. J. Clean. Prod..

[9] Catherine C., Twizerimana M. (2022). Biogas production from thermochemically pretreated sweet potato root waste. Heliyon.

[10] Annual Report on Forest and Forestry in Japan FY 2021, Forestry Agency, Ministry of Agriculture, Forestry and Fisheries, Tokyo, Japan, 2022. https://www.rinya.maff.go.jp/j/kikaku/hakusyo/r3hakusyo/attach/pdf/index-2.pdf.

[11] (2022). Overview of Japan’s energy balance flow (in Japanese). Energy White Paper.

[12] Yanagida T., Yoshida T., Kuboyama H., Jinkawa M. (2015). Relationship between feedstock price and break-even point of woody biomass power generation under FIT program. J. Jpn. Inst. Energy.

[13] Daniel-Gromke J., Rensberg N., Denysenko V., Stinner W., Schmalfuß T., Scheftelowitz M., Nelles M., Liebetrau J. (2018). Current Developments in Production and Utilization of Biogas and Biomethane in Germany. Chem. Ing. Tech..

[14] Sakamoto M., Suzuki T. (2018). Effect of pot volume on the growth of sweetpotato cultivated in the new hydroponics system. Sustain. Agric. Res..

[15] Sakamoto M., Suzuki T. (2020). Effect of nutrient solution concentration on the growth of hydroponic sweetpotato. Agronomy.

[16] Oliveira A., Dinis L.T., Santos A.A., Fontes P., Carnelossi M., Fagundes J., Oliveira-Júnior L. (2022). Particle film improves the physiology and productivity of sweet potato without affecting tuber’s physicochemical parameters. Agriculture.

[17] Li G.L., Lin Z.M., Xu Y.Q., Liu Z.H., Li H.W., Ji R.C., Luo W.B., Tang H., Qiu S.X., Qiu Y.X. (2018). Photosynthesis-light response models for varieties of sweet potato. Fujian J. Agric. Sci..

[18] Wu H.Y., Guo Q.L., Wang J.Q., Li H., Liu Q. (2019). Effects of water supply on photosynthesis and fluorescence characteristics of sweet potato [*Ipomoea batatas* (L.) Lam.] leaves and comparison of light response models. Chin. J. Eco-Agric..

[19] Oliveira A.P., Dinis L.T.R., Barbosa N.T.B., Mattos E.C., Fontes P.T.N., Carnelossi M.A.G., Fagundes J.L., Silva E.C., de Oliveira Junior L.F.G. (2021). Calcium particle films promote a photoprotection on sweet potato crops and increase its productivity. Theor. Exp. Plant Physiol..

[20] He D., Yan Z., Sun X., Yang P. (2020). Leaf development and energy yield of hydroponic sweetpotato seedlings using single-node cutting as influenced by light intensity and LED spectrum. J. Plant Physiol..

[21] Chan C.F., Lien C.Y., Lai Y.C., Huang C.L., Liao W.C. (2010). Influence of purple sweet potato extracts on the UV absorption properties of a cosmetic cream. J. Cosmet. Sci..

[22] Li M., Jang G.Y., Lee S.H., Kim M.Y., Hwang S.G., Sin H.M., Kim H.S., Lee J., Jeong H.S. (2017). Comparison of functional components in various sweet potato leaves and stalks. Food Sci. Biotechnol..

[23] Ramamoorthy P., Bheemanahalli R., Meyers S.L., Shankle M.W., Reddy K.R. (2022). Drought, low nitrogen stress, and ultraviolet-B radiation effects on growth, development, and physiology of sweetpotato cultivars during early season. Genes.

[24] Wei M., Zhang A., Li H., Tang Z., Chen X. (2015). Growth and physiological response to nitrogen deficiency and re-supply in leaf-vegetable sweetpotato (*Ipomoea batatas* Lam). HortScience.

[25] Lin K.H., Chao P.Y., Yang C.M., Chen W.C., Lo H.F., Chang T.R. (2006). The effects of flooding and drought stresses on the anti-oxidant constituents in sweet potato leaves. Bot. Stud..

[26] Laksmini N.P.L., Paramita N.L.P.V., Wirasuta I.M.A.G. (2016). In vitro and silico antioxidant activity of purified fractions from purple sweet potato ethanolic extract. Int. J. Pharm. Pharm. Sci..

[27] Tan B.C., Cline K., McCarty D.R. (2001). Localization and targeting of the VP14 epoxy-carotenoid dioxygenase to chloroplast membranes. Plant J..

[28] Lin H.H., Lin K.H., Jiang J.Y., Wang C.W., Chen C.I., Huang M.Y., Weng J.H. (2021). Comparisons between yellow and green leaves of sweet potato cultivars in chlorophyll fluorescence during various temperature regimes under high light intensities. Sci. Hortic..

[29] Alemu S.T., Roro A.G. (2020). Effect of solar ultraviolet-B plus end of day light and its exclusion on growth performance and dry weight accumulation of two sweet potato cultivars (*Ipomoea batatas* L.) on different altitudes. Int. J. Hortic. Sci. Technol..

[30] Chen Z., Gao W., Reddy K.R., Chen M., Taduri S., Meyers S.L., Shankle M.W. (2020). Ultraviolet (UV) B effects on growth and yield of three contrasting sweet potato cultivars. Photosynthetica.

[31] Dom M., Ayalew W. (2010). Effect of replacing 50% of a commercial grower feed with sweet potato silage on the performance of crossbred pigs in Papua New Guinea. J. South Pac. Agric..

[32] Sardoei S.A. (2014). Evaluation chlorophyll contents assessment on *Spathiphyllum wallisii* Regel with plant growth regulators. Int. J. Biol. Sci..

[33] Sakamoto M., Wada M., Suzuki T. (2020). Effect of partial excision of early taproots on growth and components of hydroponic carrots. Horticulturae.

[34] Sakamoto M., Komatsu Y., Suzuki T. (2021). Nutrient deficiency affects the growth and nitrate concentration of hydroponic radish. Horticulturae.

